# Effect of thyroid function on COPD exacerbation frequency: a preliminary study

**DOI:** 10.1186/2049-6958-8-64

**Published:** 2013-10-01

**Authors:** Sevinc Sarinc Ulasli, Serife Savas Bozbas, Zeynep Erayman Ozen, Berna Akinci Ozyurek, Gaye Ulubay

**Affiliations:** 1 Medical Faculty, Department of Pulmonary Diseases, Afyon Kocatepe University, Afyon, Turkey; 2 Medical Faculty, Department of Pulmonary Diseases, Baskent University, Ankara, Turkey; 3 Ankara Ataturk Chest Diseases and Chest Surgery Training and Research Hospital, Ankara, Turkey

**Keywords:** COPD, Exacerbation frequency, Hypothyroidism, Quality of life

## Abstract

**Background:**

Frequent exacerbations of chronic obstructive pulmonary disease (COPD) have negative effects on quality of life and survival. Thus, factors related to exacerbations should be determined. We aimed to evaluate the effects of thyroid function on quality of life and exacerbation frequency in COPD patients.

**Methods:**

The study population (n = 128) was divided into 3 groups (Group 1: COPD patients with hypothyroidism (n = 44); Group 2: COPD patients with normal thyroid function tests (n = 44); Group 3: Healthy subjects (n = 40)). Pulmonary function tests, maximum inspiratory pressure (MIP) and maximum expiratory pressure (MEP) measurements were performed. Quality of life questionnaire (Short Form 36, SF-36) was carried out. Patients were followed up for one year and number of exacerbations was recorded.

**Results:**

FVC, FEV_1_/FVC, and FEF _25–75%_ measurements were statistically different between group 1 and 2 (p = 0.041, p = 0.001, p = 0.009 respectively). Although MEP values were significantly different between group 1 and 2 (p = 0.006), there was no significant difference in MIP values between groups (p = 0.77). Quality of life scores in group 1 and 2 were significantly lower than control group. Exacerbation frequency was significantly higher in group 1 than in group 2 (p = 0.017). TSH values and exacerbation frequency had positive correlation (p < 0.0001; r = 0.82).

**Conclusions:**

The results of the present study suggest that thyroid function has an effect in exacerbation frequency of COPD. Decrease in exacerbation numbers with early detection of impairment in thyroid function will have positive contribution on quality of life in COPD patients.

## Background

Chronic obstructive pulmonary disease (COPD) is a respiratory disease with systemic complications, which is characterized by chronic airflow limitation due to destruction of lung parenchyma and airways [1]. Many systems including endocrine system are affected in COPD. Extrapulmonary effects of COPD and comorbidities diminish quality of life, aggravate symptoms and increase mortality [2-4].

Thyroid hormones play an important role in the regulation of thermogenesis and metabolism. Serum thyroid hormone levels change during systemic illnesses. Previous studies reported changes in thyroid hormone levels in respiratory diseases [5]. Hypothyroidism may also cause alveolar hypoventilation, decreased lung volumes, upper airway obstruction, depression in respiratory stimulus and respiratory failure. Hypoxia and decreased ventilatory response to hypercapnia have been demonstrated in patients with hypothyroidism [6,7]. Diaphragmatic dysfunction and myopathia can be seen in patients with hypothyroidism. Inspiratory and expiratory muscle strength is linearly related to the degree of hypothyroidism [8,9]. The myopathic manifestations may be related to impaired expression of myosin heavy chains IIb or to impaired neuromuscular transmission [10].

Thyroid function of COPD patients has been investigated in previous studies and different results have been found [11]. Hypoxia and hypercapnia cause destruction in sellaturcica and pituitary gland dysfunction. During the course of COPD, together with hypoxia, peripheral metabolism of thyroid function changes and thyroid hormone levels decrease in patients with very severe COPD [12,13]. Previous studies have also shown altered thyroid function during COPD exacerbations [14]. To the best of our knowledge the effects of thyroid function on acute exacerbations frequency in COPD patients have not been investigated so far.

We hypothesized that impaired thyroid function may contribute to the impaired quality of life and increase exacerbation frequency in COPD patients. Thus we aimed to investigate quality of life in COPD patients with and without hypothyroidism and to determine a relationship between acute exacerbations frequency and thyroid function.

## Methods

A prospective case control study was conducted in COPD patients. Institutional review board of our university approved the protocol and informed consent form was signed by each participant.

### Study population

COPD patients who have been regularly followed up at outpatient clinics of Pulmonary Diseases Department of Baskent University Faculty of Medicine in Ankara, Turkey, between August 2009 and December 2010 were recruited to the study. Medical history, physical examination, pulmonary function tests (PFTs), chest X-rays; thyroid function test results of all patients were recorded in order to establish the diagnosis of COPD and hypothyroidism.

Diagnosis of COPD was based on current Global Initiative for Chronic Obstructive Lung Disease (GOLD) guidelines [1]. All COPD patients were ex-smokers and stable at the time of the enrollment in the study. Patients with mild, moderate and severe obstruction were recruited to the study.

COPD patients with impaired thyroid function test results were examined in the department of endocrinology. Further diagnostic investigation was performed if needed in patients with hypothyroidism to reach an accurate diagnosis.

Patient groups (COPD patients with and without hypothyroidism) were composed of patients with similar age, gender and degree of airflow obstruction. The number of patients with severe airflow obstruction was the same in each group. Patients with severe obstruction received additional inhaled corticosteroid treatment.

Patients during acute exacerbation of COPD, and who received systemic corticosteroids, medications containing iodine, amiadorone and/or contrast material within prior two months, those who could not perform the pulmonary function tests, those with thyroid surgery, other endocrine diseases (including diabetes mellitus), neuromuscular and cardiovascular diseases, symptoms of any infections or using anti-inflammatory medications, and patients with hypoxemia were excluded from the study.

Age and gender matched control subjects were selected from healthy subjects with normal thyroid function and without any systemic diseases and smoking history.

We divided study population (n = 128) into three groups (Group 1: COPD patients with hypothyroidism (n = 44), Group 2: COPD patients with normal thyroid functions (n = 44), Group 3: Healthy subjects (n = 40)).

### Thyroid function tests

Venous blood samples were collected into blood collection tubes with red cap at 8 a.m. following an overnight fast. Thyroid stimulating hormone (TSH) (normal range: 0.35–4.0 mIU/L), free triiodothyronine (FT3) (normal range: 2.3–6.7 pmol/L) and free tetraiodothyronine (FT4) (normal range: 10.2–24.4 pmol/L) were measured with using electrochemiluminescence immunoassay (E170, Mannheim, Germany). Patients with elevated serum TSH levels (higher values than 4.0 mIU/ml) and a low serum FT4 concentration were diagnosed as primary hypothyroidism, whereas patients with normal FT4 concentration in the presence of an elevated TSH concentration were diagnosed as subclinical hypothyroidism. Patients with secondary (central) hypothyroidism had a low serum FT4 concentration and a serum TSH concentration that was not appropriately elevated. 32 COPD patients with primary hypothyroidism, 4 COPD patients with secondary hypothyroidism, and 8 COPD patients with subclinic hypothyroidism were included in the study.

### Pulmonary function tests

Pulmonary function tests were performed with a clinical spirometer (SensorMedicsVmax spectra 229, Bilthoven, The Netherlands). Maximal expiratory flow maneuver was performed by patients and control subjects. Forced expiratory volume in 1 second (FEV_1_) and forced vital capacity (FVC) values were obtained and FEV_1_/FVC was calculated. Standard PFTs including spirometry and lung volumes were evaluated according to the previously described guidelines [15]. Patients with post-bronchodilator FEV_1_/FVC <70%, and irreversible airflow obstruction were recruited to the study [16]. Post-bronchodilator FEV_1_ values were used to define disease stage according to the GOLD severity classification [1].

### Evaluation of respiratory muscle strength

Respiratory muscle strength was assessed by measuring the maximal inspiratory pressure (MIP) reflecting the strength of the diaphragm and other inspiratory muscles, and the maximal expiratory pressure (MEP) reflecting the strength of the abdominal muscles and other expiratory muscles [17]. A hand-held Micro RPM (respiratory pressure meter) was used to measure respiratory muscle strength [18]. The technician explained the procedure before the test and demonstrated the correct manoeuvre.

### Quality of life measurement

The SF-36 questionnaire was used to evaluate quality of life (QOL). This questionnaire has been previously validated for COPD patients [19,20]. The subjects’ daily routine activities, social life and exercise performance were determined based on 36 questions of this item. Main eight domains as physical function, social function, physical and emotional role limitation, mental health, pain, vitality, and general health perception were found. A computer algorithm was used to score the responses to the SF-36 [21].

### Acute exacerbation frequency

Diagnosis of an exacerbation relies on clinical presentation of the patient complaining of worsening symptoms (dyspnea, cough or sputum production) and leading to an increase in the use of maintenance medications and/or supplementation with additional medications that is beyond normal day to day variations [1]. All patients were followed up for one year after the enrollment to the study and number of exacerbations was recorded. The data regarding the exacerbation frequency were collected via regular outpatient visits every 3 months, hospitalizations, emergency room admissions and telephone calls on a thorough review of patient’s symptoms.

### Statistical analysis

The statistical analyses of our study were performed using SPSS statistical software version 20.0. The variables were investigated using visual (histograms, probability plots) and analytical methods (Kolmogorov Smirnov test) to determine the normality of distributions. The results were expressed as mean ± standard deviation and median value (min-max range). ANOVA was used to compare parameters with normal distribution among study groups (group 1, 2, and 3). Levene’s test was used to assess homogeneity of variances. P less than 0.05 was accepted as significance level. When an overall significance was observed pairwise *post hoc* tests were performed using Tukey’s test for homogenous variances and Tamhane’s T2 test for heterogenous variances. For continuos variables without normal distribution Mann–Whitney *U* test was used for the comparison of the two groups (patients with and without hypothyroidism), whereas Kruskal-Wallis test for the comparison of parameters among 3 groups. T- test was used for the comparison of parameters with normal distribution between 2 groups. The parameters affecting acute exacerbation frequency were investigated using Pearson and Spearman correlation analysis. Fisher’s exact test was used to compare disease stages in two groups.

A multiple linear regression model was used to identify independent predictors of acute exacerbation frequency. The model fit was assessed using appropriate residual and goodness of fit statistics.

## Results

Demographic data and pulmonary function test results of our study population are demonstrated in Table 1. Age, body mass index (BMI), height and weight were not different among study groups. *Post hoc* analysis showed that MEP values were significantly different between group 1 and 2, and group 2 and 3 (p = 0.006; p = 0.018 respectively). Cigarette pack/year, FEV_1_/FVC, FVC (liter and %), and FEF%_25–75_ were significantly different among the three groups (Table 1) (group 1 vs 2, group 1 vs 3, group 2 vs 3).

**Table 1:** Demographic data, pulmonary function test and SF-36 results of the study population

**PARAMETERS**	**COPD + Hypothyroidism**	**COPD**	**CONTROL**	**P**
	**Group 1**	**Group 2**	**Group 3**	
	**(n = 44)**	**(n = 44)**	**(n = 40)**	
Age (years)	64.1 ± 7.1	66.5 ± 6	64.7 ± 3.4	0.26
Gender (F/M)	22/22	22/22	20/20	1
Height (cm)	162.7 ± 7.2	166.2 ± 7.3	167 ± 8.4	0.11
Weight (kg)	78.7 ± 15.8	76.1 ± 17.1	78.5 ± 12.2	0.9
BMI (kg/m^2^)	29.73 ± 5.74	27.43 ± 5.35	27.88 ± 3.98	0.296
Cigarette pack/year	37.6 ± 22.6^**a**,**b**^	54.2 ± 26.7^**a**,**c**^	0^**bc**^	<**0**.**0001**
FEV_1_/FVC	67.2 ± 11.1^**a**,**b**^	53.2 ± 15.6^**a**,**c**^	79.5 ± 5.2^**b**,**c**^	<**0**.**0001**
FEV_1_(L)	2.1 ± 0.7^**a**^	2.01 ± 0.53^**b**^	3.6 ± 0.58 ^**a**,**b**^	<**0**.**0001**
FEV_1_(%)	83.1 ± 22.2^**a**^	77.5 ± 16.2^**b**^	116.9 ± 17.5 ^**a**,**b**^	<**0**.**0001**
FVC (L)	3.1 ± 0.9^**a**,**b**^	3.67 ± 0.9^**a**,**c**^	4.6 ± 0.8 ^**b**,**c**^	<**0**.**0001**
FVC (%)	100.1 ± 19.2^**a**^	108.9 ± 21^**b**^	121.8 ± 19.6 ^**a**,**b**^	**0**.**003**
FEF_25–75_ (%)	47.5 ± 27.4^**a**,**b**^	29.6 ± 12.3^**a**,**c**^	95.1 ± 21.9 ^**b**,**c**^	<**0**.**0001**
TLC (%)	107.2 ± 19.5	105.3 ± 19	113.9 ± 15.9	0.22
TLC (L)	5.9 ± 1.4	6.5 ± 1.5	6.55 ± 1.03	0.32
MIP (cmH_2_O)	69.6 ± 30.7	67.3 ± 17.5	74.05 ± 24.3	0.673
MEP (cmH_2_O)	25.6 ± 9.1^**a**,**b**^	34.9 ± 11.5^**a**,**c**^	26.7 ± 6.4^**b**,**c**^	**0**.**004**
General health	35 (0–80)^**a**^	45 (10–70)^**b**^	69.5 (40–82)^**a**,**b**^	<**0**.**0001**
Physical functioning	52.5 (0–100) ^**a**^	62.5 (25–95) ^**b**^	90 (50–100) ^**a**,**b**^	<**0**.**0001**
Physical Role limitation	25 (0–100) ^**a**^	25 (0–100) ^**b**^	100(50–100)^**a**,**b**^	<**0**.**0001**
Emotional Role limitation	33 (0–100) ^**a**^	33 (0–100) ^**b**^	66.6(0–100) ^**a**,**b**^	<**0**.**0001**
Social functioning	50 (13–100) ^**a**^	56 (0–100) ^**b**^	87.5(38100)^**a**,**b**^	<**0**.**0001**
Pain	60 (0–100) ^**a**^	85 (20–100) ^**b**^	100(40–100)^**a**,**b**^	**0**.**029**
Vitality	52.5 (10–85) ^**a**^	57.5 (20–92) ^**b**^	80.5(45100)^**a**,**b**^	**0**.**001**
Mental health	52.5 (12–80) ^**a**^	58 (15–90) ^**b**^	72 (50–95) ^**a**,**b**^	<**0**.**0001**

Data are expressed as mean ± SD for parameters with normal distribution and median for parameters with skewed distribution (range min-max).

Where P is significant, values within a row with the same superscript letter are significantly different.

BMI, Body mass index; COPD, Chronic obstructive pulmonary disease; F/M, Female/Male; FEF_25–75_%, Forced expiratory flow at 25–75%; FEV_1_, Forced expiratory volume at 1 second; FVC, Forced vital capacity; MEP, Maximal expiratory pressure; MIP, Maximal inspiratory pressure; SF-36, Short form-36; TLC, Total lung capacity.

We also compared pulmonary function test results of group 1 and 2 and did not find statistically significant difference in terms of FEV_1_ (L and %) (p = 0.637, p = 0.339, respectively).

FVC (litre), FEV_1_/FVC, FEF%_25–75_ (litre/sec) were significantly different between group 1 and 2 (p = 0.041, p = 0.001, p = 0.009, respectively). We found a significant difference in MEP values between group 1 and 2 (p = 0.006), but not in MIP values (p = 0.77). TSH values were significantly different between group 1 and 2 (p = 0.04) (Table 2). Disease stage of patients in group 1 and 2 was not different (p = 0.169) (Table 3).

**Table 2:** Pulmonary function tests, muscle strength, thyroid function tests, SF-36 results and acute exacerbation frequency of COPD patients with and without hypothyroidism (Group 1 and 2)

**PARAMETERS**	**COPD + Hypothyroidism**	**COPD**	**P**
	**Group 1**	**Group 2**	
	**(n = 44)**	**(n = 44)**	
FEV_1_/FVC	67.2 ± 11.1	53.2 ± 15.6	0.001
FEV_1_(L)	2.1 ± 0.7	2.01 ± 0.53	0.637
FEV_1_(%)	83.1 ± 22.2	77.5 ± 16.2	0.339
FVC (L)	3.1 ± 0.9	3.67 ± 0.9	0.041
FVC (%)	100.1 ± 19.2	108.9 ± 21	0.15
FEF_25–75_ (%)	47.5 ± 27.4	29.6 ± 12.3	0.009
TLC (%)	107.2 ± 19.5	105.3 ± 19	0.76
TLC (L)	5.9 ± 1.4	6.5 ± 1.5	0.306
MIP (cmH_2_O)	69.6 ± 30.7	67.3 ± 17.5	0.77
MEP (cmH_2_O)	25.6 ± 9.1	34.9 ± 11.5	0.006
TSH (mIU/mL)	4.3 (0.1–16.8)	0.8(0.3-3.6)	0.04
FT4 (pmol/L)	2.15 ± 3.27	2.36 ± 3.56	0.87
FT3 (pmol/L)	2.37 ± 0.77	2.73 ± 0.84	0.27
Acute exacerbation frequency (/years)	1.5 ± 0.85	0.86 ± 0.83	0.017
General health	35 (0–80)	45 (10–70)	0.383
Physical functioning	52.5 (0–100)	62.5 (25–95)	0.391
Physical role limitation	25 (0–100)	25 (0–100)	0.637
Emotional role limitation	33 (0–100)	33 (0–100)	0.608
Social functioning	50 (13–100)	56 (0–100)	0.972
Pain	60 (0–100)	85 (20–100)	0.179
Vitality	52.5 (10–85)	57.5 (20–92)	0.548
Mental health	52.5 (12–80)	58 (15–90)	0.249

Data are expressed as mean ± SD for parameters with normal distribution and median for parameters with skewed distribution (range min-max).

COPD, Chronic obstructive pulmonary disease; FEF_25–75_%, Forced expiratory flow at 25–75%; FEV_1_, Forced expiratory volume at 1 second; FT3, Free triiodothyronine; FT4, Free tetraiodothyronine; FVC, Forced vital capacity; MEP, Maximal expiratory pressure; MIP, Maximal inspiratory pressure; SF-36, Short form-36; TSH, Thyroid stimulating hormone.

**Table 3:** Comparison of group 1 and 2 in terms of disease severity

**Disease stage**	**COPD + Hypothyroidism**	**COPD**	**P**
	**Group 1**	**Group 2**	
	**(n = 44)**	**(n = 44)**	
STAGE 1	30	22	0.169
STAGE 2	10	18	
STAGE 3	4	4	

COPD, Chronic obstructive pulmonary disease.

SF 36 scores in group 1 and 2 were significantly lower than in control group (Table 1). There were no significant differences between Group 1 and 2 in terms of SF 36 scores (Tables 1, 2). We found positive significant correlations between FEV_1_ (L) and scores of SF-36 domains (FEV_1_ with physical activity p < 0.0001, r = 0.582; FEV_1_ with physical role limitation p < 0.0001, r = 0.488; FEV_1_ with general health p < 0.0001 r = 0.534; FEV_1_ with vitality p < 0.0001 r = 0.434; FEV_1_ with social functioning p < 0.0001, r = 0.534; FEV_1_ with emotional role limitation p = 0.001 r = 0.412; FEV_1_ with mental health p = 0.001, r = 0.398).

Acute exacerbation frequency of group 1 was significantly higher than that of group 2 (1.5 ± 0.85 and 0.86 ± 0.83 respectively; p = 0.017) (Table 2).

When we evaluated the relationships between TSH values and demographic data and SF 36 domains, we found significant relationships between TSH and BMI and mental health (p = 0.033, r = −0.323; p = 0.037, r = −0.315; respectively). There were no significant correlations between TSH and MIP, MEP, FEV_1_ L and FVC L (p = 0.146, r = −0.228; p = 0.117, r = −0.246; p = 0.906, r = −0.018; p = 0.405, r = −0.129; respectively).

Acute exacerbation frequency was not correlated with MIP and MEP values (p = 0.51, r = −0.103; p = 0.167, r = −0.214 respectively). FVC L, FVC%, FEV_1_ L, FEV_1_% were negatively correlated with exacerbation frequency (p = 0.008, r = −0.391; p = 0.002, r = −0.448; p = 0.01, r = 0.380; p = 0.042, r = −0.304 respectively).

**Figure 1:**
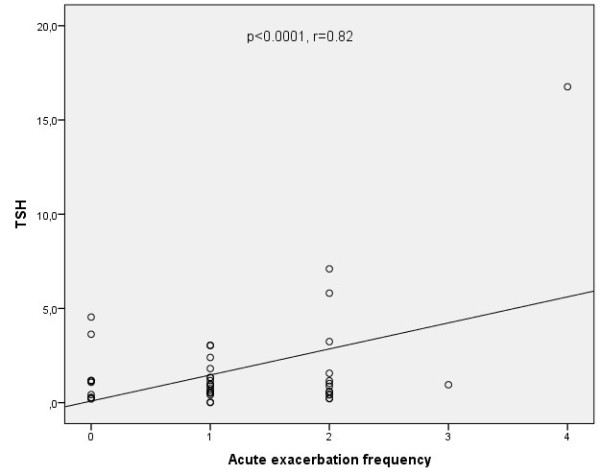
Relationship between thyroid stimulating hormone (TSH) levels and acute exacerbation frequency of COPD.

A positive significant relationship between acute exacerbation frequency and TSH values was found (p < 0.0001; r = 0.82) (Figure 1). Multiple linear regression model was used to determine the contributing factors to exacerbation frequency. Only TSH was found to be significantly associated with acute exacerbation frequency (p < 0.0001) (Table 4).

**Table 4:** Multiple linear regression model for exacerbation frequency

**Multiple linear regression model for exacerbation**				
**frequency by TSH, FEV**_**1 **_**(L and %) and FVC (L and %)**				
	**Coefficient B value**	**Standard error**	**t**	**P**
Constant	0.611	1.51	0.403	0.689
TSH levels	0.918	0.102	9.02	<0.0001
FEV_1_ (L)	−3.402	2.74	−1.23	0.223
FEV_1_(%)	0.099	0.076	1.303	0.201
FVC (L)	1.39	1.59	0.874	0.388
FVC (%)	−0.061	0.056	−1.089	0.283

COPD, Chronic obstructive pulmonary disease; FEV_1_: Forced expiratory volume at 1 second; FVC, Forced vital capacity; TSH, Thyroid stimulating hormone.

## Discussion

To our knowledge, the present study is the first one investigating quality of life and exacerbation frequency in COPD patients with and without hypothyroidism and the relationships between thyroid functions and exacerbation frequency in COPD patients.

Dyspnea, exercise limitation, depressed psychological mood, comorbidities and exacerbations are main factors affecting quality of life in COPD patients [22]. Increased perception of dyspnea decreases physical activity and dyspnea aggravates during limited physical activities so as a vicious cycle is formed. Evaluation of quality of life in COPD patients with SF-36 questionnaire is an easy and helpful method already demonstrated in previous studies [23,24]. We determined significantly decreased scores of SF-36 domains in COPD patients with and without hypothydroidism than in control subjects and FEV_1_ values were significantly correlated with SF-36 scores in accordance with the previous studies [25,26].

In a recent population based study thyroid hormone status and health related quality of life were investigated and scores of subjects with suppressed TSH values or markedly elevated TSH values were not significantly lower than those of subjects with normal or mildly elevated TSH values [27]. However, to our knowledge, up until our study the quality of life in COPD patients with and without hypothyroidism has not been evaluated. In the present study SF-36 scores of COPD patients with and without hypothyroidism did not differ significantly. Therefore we can conclude that hypothyroidism is not a factor affecting quality of life in COPD patients. This result should be addressed in the future prospective investigations with larger sample size of COPD patients.

In several diseases, the evaluation of respiratory muscle strength is very useful. It is known that a reduction of MIP and MEP has been associated with several neuromuscular diseases, but it is also possible to detect decreased values of MIP and MEP in COPD patients [28]. Malnutrition, muscular atrophy, steroid-induced myopathy, pulmonary hyperinflation and reduced blood flow to the respiratory muscles are contributing factors to respiratory muscle weakness in COPD patients [29-31]. Diaphragmatic dysfunction in hypothyroidism, and inverse relationship between TSH and inspiratory and expiratory muscles’ length have been previouslyreported [7-9]. In the present study MEP values were significantly lower in patients with hypothyroidism than in those without it. This result confirms the adverse effects of hypothyroidism on expiratory muscles. However we did not find a significant difference in MIP values between patients with and without hypothyroidism and no significant correlation between MIP and MEP values and thyroid function. These results might be due to the characteristics of our study population as we did not include patients with very severe COPD, with hypoxemia and neuromuscular disorders, or those who have received systemic corticosteroids. Besides, similar FEV_1_ values and disease stages of COPD patients with and without hypothyroidism may affect these outcomes.

Exacerbations of COPD are important events in the course of disease as exacerbations negatively affect quality of life, accelerate the decline of pulmonary function, and are associated with higher socioeconomic costs and mortality [1,32]. Development of strategies to prevent exacerbations is an important goal in COPD. In the present study we determined that exacerbation frequency in COPD patients with hypothyroidism was significantly higher than in COPD patients without hypothyroidism and we detected a significant relationship between TSH values and frequency of acute exacerbations. Moreover, we strengthened our hypothesis with linear regression analysis, because we found that the only significant determinant of exacerbation frequency was serum TSH levels in our study population.

### Limitations

The limitation of our study was failure to convince higher number of patients to participate in the study. Further studies with larger sample sizes are needed to confirm and explore the findings of the present study.

## Conclusions

In conclusion, our preliminary study demonstrates a significant relationship between TSH levels and COPD exacerbation frequency which suggests that the detection of impairment in thyroid function can decrease exacerbation number and improve quality of life in COPD patients.

## Availability of supporting data

The data set supporting the results of the present study is present within the article.

## Competing interest

The authors declare that they have no competing interests.

## Authors’ contributions

SSU designed the study, conducted the study, collected and analyzed data, and prepared the manuscript. SSB helped in analysis of data and prepared the manuscript. ZEO and BAO helped in the conduction of the study and collection of data. GU helped in the conduction of the study, and preparation of the manuscript. All authors read and approved the final manuscript.

## Authors’ information

SSU: M.D., Assistant Professor in Afyon Kocatepe University, Medical Faculty, Department of Pulmonary Diseases, Afyon, Turkey.

SSB: M.D., Associate Professor, in Baskent University, Medical Faculty, Department of Pulmonary Diseases, Ankara, Turkey.

ZEO: M.D., Physician, in Baskent University, Medical Faculty, Department of Pulmonary Diseases, Ankara, Turkey.

BAO: M.D., Physician, in Ankara Ataturk Chest Diseases and Chest Surgery Training and Research Hospital, Ankara, Turkey.

GU: M.D., Professor, in Baskent University, Medical Faculty, Department of Pulmonary Diseases, Ankara, Turkey.

## References

[B1] Global strategy for the diagnosis, management and prevention of chronic obstructive pulmonary disease (GOLD, updated 2013) Available at: http://www.goldcopd.org 10.1164/rccm.201204-0596PP 22878278

[B2] Fumagalli G Fabiani F Forte S Napolitano M Marinelli P Palange P Pentassuglia A Carlone S Sanguinetti CM INDACO project: a pilot study on incidence of comorbidities in COPD patients referred to pneumology units Multidiscip Resp Med 2013 8 28 10.1186/2049-6958-8-28 PMC3637139 23551874

[B3] Vanfleteren LE Spruit MA Groenen M Gaffron S vanEmpel VP Bruijnzeel PL Rutten EP Roodt J Wouters EF Franssen FM Clusters of comorbidities based on validated objective measurements and systemic inflammation in patients with chronic obstructive pulmonary disease Am J Respir Crit Care Med 2013 187 7 728 735 10.1164/rccm.201209-1665OC 23392440

[B4] Thomsen M Dahl M Lange P Vestbo J Nordestgaard BG Inflammatory biomarkers and comorbidities in chronic obstructive pulmonary disease Am J Respir Crit Care Med 2012 186 10 982 988 10.1164/rccm.201206-1113OC 22983959

[B5] Verleden GM Demedts MG Westhovens R Thomeer M Pulmonary manifestations of systemic diseases EurRespir Monogr 2006 34 234 252

[B6] Zwillich CW Pierson DJ Hofeldt FD Lufkin EG Weil JV Ventilatory control in myxedema and hypothyroidism N Engl J Med 1975 292 662 665 10.1056/NEJM197503272921302 1113761

[B7] Saaresranta T Polo O Hormones and breathing Chest 2002 122 2165 2182 10.1378/chest.122.6.2165 12475861

[B8] Siafakas NM Salesiotou V Filaditaki V Tzanakis N Thalassinos N Bouros D Respiratory muscle strength in hypothyroidism Chest 1992 102 189 194 10.1378/chest.102.1.189 1623751

[B9] Datta D Scalise P Hypothyroidism and failure to wean in patients receiving prolonged mechanical ventilation at a regional weaning center Chest 2004 126 1307 1312 10.1378/chest.126.4.1307 15486397

[B10] Laghi F Tobin MJ Disorders of the respiratory muscles Am J Respir Crit Care Med 2003 168 10 48 10.1164/rccm.2206020 12826594

[B11] Creutzberg EC Casaburi R Endocrinological disturbances in chronic obstructive pulmonary disease Eur Respir J 2003 22 76 80 10.1183/09031936.03.00004610 14621109

[B12] Gow SM Seth J Beckett GJ Douglas G Thyroid function and endocrine abnormalities in elderly patients with severe chronic obstructive pulmonary disease Thorax 1987 42 520 525 10.1136/thx.42.7.520 3125626 PMC460817

[B13] Dimopoulou I Ilias I Mastorakos G Mantzos E Roussos C Koutras DA Effects of severity of chronic obstructive pulmonary disease on thyroid function Metabolism 2001 50 1397 1401 10.1053/meta.2001.28157 11735083

[B14] Soyyigit S Curgunlu A Tufekci IB Tutluoglu B The incidence of sick euthyroid syndrome in acute exacerbation of COPD Solunum 2004 6 14 17

[B15] Pellegrino R Viegi G Brusasco V Crapo RO Burgos F Casaburi R Coates A van der Grinten CP Gustafsson P Hankinson J Jensen R Johnson DC MacIntyre N McKay R Miller MR Navajas D Pedersen OF Wanger J Interpretative strategies for lung function tests Eur Respir J 2005 26 5 948 968 10.1183/09031936.05.00035205 16264058

[B16] Miller MR Hankinson J Brusasco V Burgos F Casaburi R Coates A Crapo R Enright P van der Grinten CP Gustafsson P Jensen R Johnson DC MacIntyre N McKay R Navajas D Pedersen OF Pellegrino R Viegi G Wanger J ATS/ERS Task Force Standardisation of spirometry Eur Respir J 2005 26 2 319 338 10.1183/09031936.05.00034805 16055882

[B17] American Thoracic Society/European Respiratory Society ATS/ERS Statement on respiratory muscle testing Am J Respir Crit Care Med 2002 166 4 518 624 15 12186831 10.1164/rccm.166.4.518

[B18] Dimitriadis Z Kapreli E Konstantinidou I Oldham J Strimpakos N Test/retest reliability of maximum mouth pressure measurements with the MicroRPM in healthy volunteers Respir Care 2011 56 6 776 782 10.4187/respcare.00783 21310113

[B19] Ferrer M Alonso J Morera J Marrades RM Khalaf A Aguar MC Plaza V Prieto L Antó JM Chronic obstructive pulmonary disease stage and health- related quality of life. The Quality of Life of Chronic Obstructive Pulmonary Disease Study Group Ann Intern Med 1997 127 1072 1079 10.7326/0003-4819-127-12-199712150-00003 9412309

[B20] Ulubay G Ulasli SS Akinci B Gorek A Akcay S Assessment of relation among emotional status, pulmonary function test, exercise performance, and quality of life in patients with COPD Tuberk Toraks 2009 57 2 169 176 19714508

[B21] Ware JE Sherbourne CD The MOS 36-item Short- Form Health Survey (SF-36). I. Conceptual framework and item selection Med Care 1992 30 473 483 10.1097/00005650-199206000-00002 1593914

[B22] Burgel PR Escamilla R Perez T Carré P Caillaud D Chanez P Pinet C Jebrak G Brinchault G Court-Fortune I Paillasseur JL Roche N INITIATIVES BPCO Scientific Committee Impact of comorbidities on COPD-specific health-related quality of life Respir Med 2013 107 2 233 241 10.1016/j.rmed.2012.10.002 23098687

[B23] Pickard AS Yang Y Lee TA Comparison of health-related quality of life measures in chronic obstructive pulmonary disease Health Qual Life Outcomes 2011 18 9 26 21501522 10.1186/1477-7525-9-26 PMC3096892

[B24] Akinci AC Pinar R Demir T The relation of the subjective dyspnoea perception with objective dyspnoea indicators, quality of life and functional capacity in patients with COPD J Clin Nurs 2013 22 7–8 969 976 22765349 10.1111/j.1365-2702.2012.04161.x

[B25] Soyyiğit S Erk M Güler N Kilinç G The value of SF-36 questionnaire for the measurement of life quality in chronic obstructive pulmonary disease Tuberk Toraks 2006 54 3 259 266 17001544

[B26] Ståhl E Lindberg A Jansson SA Rönmark E Svensson K Andersson F Löfdahl CG Lundbäck B Health-related quality of life is related to COPD disease severity Health Qual Life Outcomes 2005 3 56 10.1186/1477-7525-3-56 16153294 PMC1215504

[B27] Klaver EI van Loon HC Stienstra R Links TP Keers JC Kema IP Muller Kobold AC Van der Klauw MM Wolffenbuttel BH Thyroid hormone status and health-related quality of life in the lifeLines cohort study Thyroid 2013 23 1066 1073 doi:10.1089/thy.2013.0017 10.1089/thy.2013.0017 23530992 PMC3770241

[B28] Terzano C Ceccarelli D Conti V Graziani E Ricci A Petroianni A Maximal respiratory static pressures in patients with different stages of COPD severity Respir Res 2008 9 8 10.1186/1465-9921-9-8 18208602 PMC2244619

[B29] Rochester DF Malnutrition and the respiratory muscles Clin Chest Med 1986 7 91 99 3514092

[B30] Openbrier DR Irwin MM Rogers RM Gottlieb GP Dauber JH Van Thiel DH Pennock BE Nutritional status and lung function in patients with emphysema and chronic bronchitis Chest 1983 83 17 22 10.1378/chest.83.1.17 6848330

[B31] Decramer M Stas KJ Corticosteroids induced myopathy involving respiratory muscles in patient with chronic obstructive pulmonary disease and asthma Am Rev Respir Dis 1992 146 800 802 10.1164/ajrccm/146.3.800 1519868

[B32] Donaldson GC Seemungal TA Bhowmik A Wedzicha JA Relationship between exacerbation frequency and lung function decline in chronic obstructive pulmonary disease Thorax 2002 57 10 847 852 10.1136/thorax.57.10.847 12324669 PMC1746193

